# Quantification of Osteoclasts in Culture, Powered by Machine Learning

**DOI:** 10.3389/fcell.2021.674710

**Published:** 2021-05-25

**Authors:** Edo Cohen-Karlik, Zamzam Awida, Ayelet Bergman, Shahar Eshed, Omer Nestor, Michelle Kadashev, Sapir Ben Yosef, Hussam Saed, Yishay Mansour, Amir Globerson, Drorit Neumann, Yankel Gabet

**Affiliations:** ^1^Blavatnik School of Computer Science, Tel Aviv University, Tel Aviv, Israel; ^2^Department of Cell and Developmental Biology, Sackler Faculty of Medicine, Tel Aviv University, Tel Aviv, Israel; ^3^Department of Anatomy and Anthropology, Sackler Faculty of Medicine, Tel Aviv University, Tel Aviv, Israel

**Keywords:** osteoclasts, automatic quantification of osteoclasts, machine learning, object detection, deep learning, convolutional neural network (CNN), deep neural networks (DNN), artificial intelligence

## Abstract

*In vitro* osteoclastogenesis is a central assay in bone biology to study the effect of genetic and pharmacologic cues on the differentiation of bone resorbing osteoclasts. To date, identification of TRAP+ multinucleated cells and measurements of osteoclast number and surface rely on a manual tracing requiring specially trained lab personnel. This task is tedious, time-consuming, and prone to operator bias. Here, we propose to replace this laborious manual task with a completely automatic process using algorithms developed for computer vision. To this end, we manually annotated full cultures by contouring each cell, and trained a machine learning algorithm to detect and classify cells into *preosteoclast* (TRAP+ cells with 1–2 nuclei), *osteoclast type I* (cells with more than 3 nuclei and less than 15 nuclei), and *osteoclast type II* (cells with more than 15 nuclei). The training usually requires thousands of annotated samples and we developed an approach to minimize this requirement. Our novel strategy was to train the algorithm by working at “patch-level” instead of on the full culture, thus amplifying by >20-fold the number of patches to train on. To assess the accuracy of our algorithm, we asked whether our model measures osteoclast number and area at least as well as any two trained human annotators. The results indicated that for osteoclast type I cells, our new model achieves a Pearson correlation (r) of 0.916 to 0.951 with human annotators in the estimation of osteoclast number, and 0.773 to 0.879 for estimating the osteoclast area. Because the correlation between 3 different trained annotators ranged between 0.948 and 0.958 for the cell count and between 0.915 and 0.936 for the area, we can conclude that our trained model is in good agreement with trained lab personnel, with a correlation that is similar to inter-annotator correlation. Automation of osteoclast culture quantification is a useful labor-saving and unbiased technique, and we suggest that a similar machine-learning approach may prove beneficial for other morphometrical analyses.

## Introduction

Bone is a highly dynamic tissue that undergoes continuous remodeling throughout life, in a process involving the concerted actions of monocyte-derived osteoclasts that resorb mineralized tissue, and mesenchymal osteoblasts that deposit new bone (Teitelbaum, 2007; Clarke, 2008; Karsenty et al., 2009).

Bone mass is carefully maintained by a tight coordination of the activity of the bone-resorbing osteoclasts and bone-forming osteoblasts (Boyle et al., 2003; Teitelbaum and Ross, 2003; Takayanagi, 2007; Zaidi, 2007), and an imbalance between their activities results in skeletal pathologies such as osteoporosis (Bruzzaniti and Baron, 2006; Novack and Teitelbaum, 2008). Compounds demonstrating any ability to modulate the balance between osteoclasts and osteoblasts are thus of great interest in treating such diseases.

Osteoclast precursor differentiation to functionally active multinucleated osteoclasts depends on administration of macrophage colony stimulating factor (M-CSF) and receptor activator for nuclear factor kappa B ligand (RANKL) (Teitelbaum, 2000).

This process, called osteoclastogenesis can be examined *in vitro* using a well-established and commonly used assay in which bone marrow derived macrophages are cultured with M-CSF and RANKL (Marino et al., 2014). During differentiation, the cells acquire a higher expression of tartrate-resistant acid phosphatase (TRAP) (Minkin, 1982) and fuse together to become multinucleated cells (Hata et al., 1992). Osteoclasts are commonly defined *in vitro* as TRAP positive (using specific staining) multinucleated cells. This assay is important for the experimental screening of therapeutic candidates targeting osteoclastogenesis and bone resorption.

The current gold standard method for quantifying osteoclast formation in culture is based on manual counting of TRAP-positive, multinucleated (≥3 nuclei) cells visualized under the microscope, which is both a subjective and a time-consuming process of evaluation (Marino et al., 2014). To overcome this significant drawback, we now present an unbiased high-throughput approach that enables fast and accurate measurement of osteoclast number and area for more efficient screening of potential therapeutic agents in bone biology. With the development of machine learning techniques, image classification and object detection applications are becoming more accurate and robust. As a result, machine learning based methods are being applied in a wide range of fields. The use of computer vision algorithms for the analysis of microscopic images has received growing interest in recent years. Such methods have been used for tasks such as detecting cell membranes (Ciresan et al., 2012), detecting cell nuclei (Xue and Ray, 2017), and segmenting cells (Ronneberger et al., 2015). This success motivated us to develop such an approach for osteoclasts. We note that existing tools cannot be directly applied to our setting due to its unique characteristics (see below).

Here, we report the development of an artificial intelligence-based object detection method designed to identify, classify, and quantify osteoclasts in cultures.

To our knowledge, there is no publicly- or commercially available software dedicated to *in vitro* osteoclast detection and evaluation. Previously developed AI-based approaches for cell detection are not applicable for this purpose due to its unique characteristics; the structure of osteoclasts is different from other cells (multinucleated TRAP-stained cells); collecting data for training an AI system dedicated to osteoclasts is time consuming (i.e., annotating a single culture takes several hours to annotate), thus limiting the number of examples that can be obtained in a timely manner. In order to overcome these challenges, we describe a method we developed to train our model on TRAP-stained osteoclast cultures and thereby increase the effective size of the training data.

For the purpose of training and evaluating our algorithm, we manually annotated 11 full osteoclast cultures containing thousands of cells. Osteoclast precursor cultures were treated with M-CSF and RANKL to induce differentiation. Images resulting from the TRAP staining were used to train the system to identify and count multinucleated TRAP+ osteoclasts, and to further validate and generalize the method. The trained model was tested on images that were not included in the training phase.

## Materials and Methods

### Materials

Minimum Essential Medium α (Alpha-MEM) and fetal bovine serum (FBS), referred to here as “Standard medium,” were purchased from Rhenium (Modiin, Israel), and culture plates were from Corning (New York, NY, United States). As a source of M-CSF, we used supernatant from CMG 14–12 cells, containing 1.3μg/*ml* M-CSF (Takeshita et al., 2000). RANKL was purchased from R&D Systems, Minneapolis, MN, United States.

### Animals

Female wild type mice of the inbred strain C57BL/6J-RccHsd, aged 8–12 weeks were purchased from Envigo (Israel) and housed at the Tel-Aviv University animal facility. These mice were used for the generation of bone marrow derived macrophages (BMDM). Animal care and all procedures were in accordance with and with the approval of the Tel Aviv University Institutional Animal Care and Use Committee (Permit number 01-19 -032).

### Cell Culture

Bone marrow cells were harvested from femurs and tibias of 8- to 12-week-old female mice. Cells were seeded on tissue-culture treated plates in standard medium (alpha-MEM supplemented with 10% fetal bovine serum). On the following day, non-adherent cells were seeded in non–tissue culture-treated plates in standard medium supplemented with 100 ng/ml M-CSF, which induces cell proliferation and differentiation into preosteoclasts.

For the osteoclastogenesis assay, preosteoclasts were plated in 96-well plates (8,000 cells per well) with standard medium supplemented with 20 ng/ml M-CSF and 50 ng/ml RANKL, replaced every 2 days. On the 4th day, cells were stained using a TRAP staining kit (Sigma-Aldrich, St. Louis, MO, United States), and multinucleated (≥3 nuclei) TRAP-positive cells were defined as osteoclasts. Images were acquired at a magnification of ×4 (Evos FLC, Life Technologies, MS, United States) (Hiram-Bab et al., 2015). An open-source graphical image annotation tool was used to measure osteoclast number and surface area from a single operator manual tracing for each well as previously described (Wada, 2016).

### Machine Learning Pipeline

Machine learning algorithms are used in a wide range of domains and underlie many technologies such as speech recognition, image understanding, machine translation, fraud detection, and face recognition.

To understand how machine learning algorithms work, we can consider an image categorization problem where the task of the model is to label an image with the name of the object in the image. Formally, the goal is to map from an input image X to an output label Y. The learning model is then simply a function from X to Y. In order to learn this function, one collects a “training” dataset of X-Y pairs (i.e., images and the corresponding correct label), and the learning process seeks a function that fits this training data well.

**FIGURE 1:**
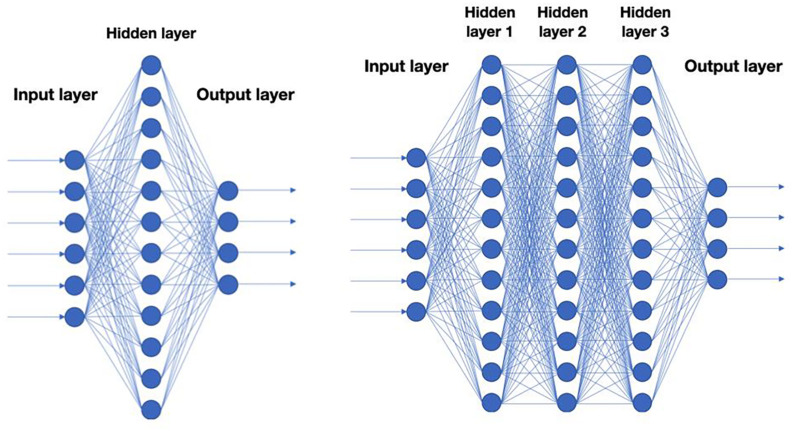
Schematic representation of neural nets. Each circle corresponds to a *neuron* in a neural net. A neuron is typically a linear function of the previous layer followed by a non-linearity. Left: A neural net with a single hidden layer. Right: Deep neural net with 3 hidden layers.

A key question is of course what type of functions to use when mapping X to Y. In recent years, functions mimicking neural networks have been found to work particularly well for a wide range of problems (Figure 1). This approach is also known as deep learning because it involves a multi-layered computation process.

**FIGURE 2:**
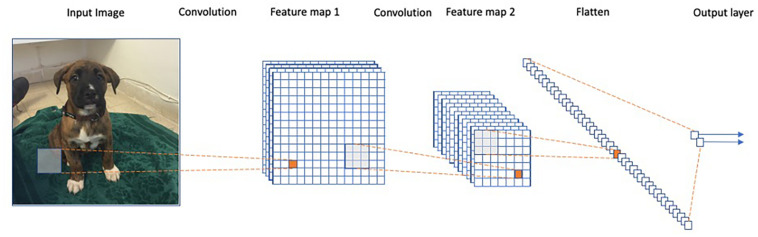
Representation of a convolutional neural network. Each convolution operation consists of applying the same filters across the image to obtain a feature map in the following layer. The final iteration produces a fully connected layer similar to a regular neural net.

Arguably, the most striking successes of deep learning to date are in the field of computer vision, where the goal is to develop algorithms that perform a semantic analysis of images, in a manner similar to the human visual system. This field has undergone a revolution since the introduction of the AlexNet architecture (Krizhevsky et al., 2012), and many other more advanced architectures since (Simonyan and Zisserman, 2014; Szegedy et al., 2015; He et al., 2016). AlexNet is an example of a so-called Convolutional Neural Network (CNN) model. These models use specific connectivity patterns between layers to form an architecture that utilizes the spatial structure within images (Figure 2).

### Object Detection

Our goal in this study was to automatically evaluate the number and area of osteoclasts in TRAP-stained cultures. To perform this task, we need to detect the cells in an image and then predict the total number of cells and the ratio between the area covered by cells and the size of the culture. This problem is closely related to the machine vision problem of *Object Detection*, which is designed to locate and correctly classify objects in images.

**FIGURE 3:**
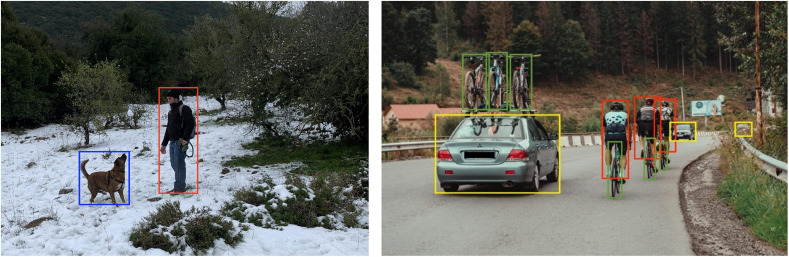
Output of SSD trained on Pascal VOC. The algorithm detects and wraps the objects with bounding boxes as well as correctly classifying them into the relevant class (the classification is depicted by the bounding box color: red corresponds to “Person” class, blue to “Dog” class, yellow to “Car” class, and green to “Bicycle” class).

Object detection is a key task in computer vision and has been the focus of much research in recent years (Girshick et al., 2014; Girshick, 2015; Ren et al., 2015). Algorithms developed for this purpose are at the heart of many evolving technologies including autonomous vehicles (Menze and Geiger, 2015) and robotics (Jia et al., 2011). The purpose of object detection pipelines is to locate and correctly classify objects in images (see Figure 3). The architecture used for objective detection is a variant of the CNNs described above, but with additional mechanisms for finding multiple objects in an image and providing suitable bounding boxes and visual categories.

**FIGURE 4:**
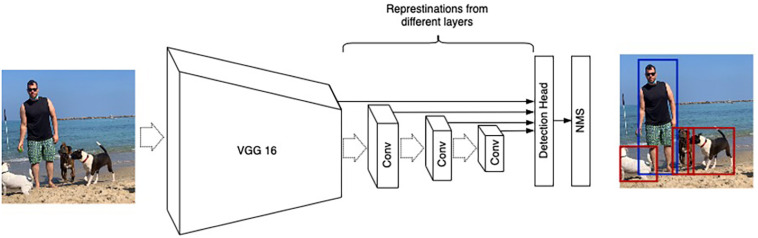
SSD architecture illustration. VGG16 (Simonyan and Zisserman, 2014) is used as a base network. Detection heads are connected to numerous convolution layers (denoted as “Conv”) to account for different resolutions.

This study is based on the Single Shot Detection (SSD) architecture (Liu et al., 2016), which has proven useful for detecting small objects (Lam et al., 2018). SSD consists of two parts: (1) A *backbone* Convolutional Neural Network that extracts features from an input image, and (2) several convolutional layers with detection heads that output bounding boxes and the corresponding class followed by Non-Maximum Suppression (NMS) to filter overlapping bounding boxes (Felzenszwalb et al., 2009; Girshick et al., 2014; Girshick, 2015; Figure 4).

The most common use of SSD is for object detection in natural images. As a training set for such SSD models, one uses large datasets of natural images that have been annotated with bounding boxes for visual objects, as well as their visual labels such as the Pascal VOC and MSCOCO datasets (Lin et al., 2014; Everingham et al., 2015).

Here our focus is quite different, as we are interested in detecting images in cell cultures, and thus models trained on natural images are not directly applicable. Instead, the approach we took here is to collect a new dataset of annotated cell images and use this to train a new model. This process will be described in more detail.

### Data Collection

One of the main challenges in utilizing deep learning algorithms in various domains is the need for vast amounts of labeled data for training. Manually labeling data for object detection is a time consuming and expensive process due to the need to annotate each bounding box. In addition, the human annotator may require specific training. In this section we describe the data collection process and considerations.

**FIGURE 5:**
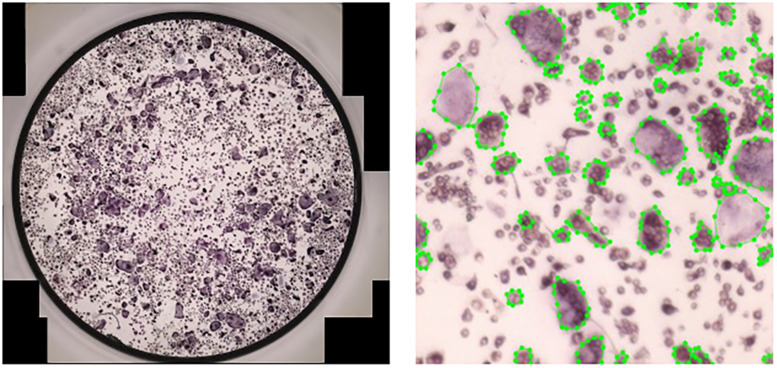
Manual annotation of osteoclasts in cultures. Left: A full culture to be annotated. Right: A crop of size $\frac{1}{16}\times \frac{1}{16}$ of a fully annotated culture.

For data annotation, we used an open-source annotation tool (Wada, 2016) to mark polygons around the cells (Figure 5). Marking polygons is more time-consuming than bounding boxes but it allows for a more versatile application such as running algorithms that quantify the contour of cells and segmentation (not tested in the scope of the current study).

**FIGURE 6:**
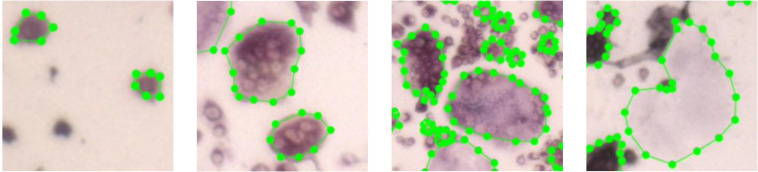
Images of the four different cell types. The first three images depict cells with different number of nuclei. The rightmost cell is a ghost cell.

We divided the cells into four different types (Figure 6): (1) *preosteoclast* – TRAP+ cells with 1–2 nuclei; (2) *osteoclast type I* – cells with more than (≥) 3 nuclei and less than 15 nuclei; (3) *osteoclast type II* – cells with more than (≥) 15 nuclei; (4) *ghost cells* – vanished cells which are distinguishable by their silhouette.

Full cultures may contain over 500 cells (Figure 5) and are hard to label accurately. To produce meaningful annotations, we divided each culture into equally sized regions, which are each annotated as separate images. This introduces a trade-off between the annotation speed and the precision of the annotations. We found that dividing each culture well to 16 regions, produced accurate annotations in a reasonable time.

**FIGURE 7:**
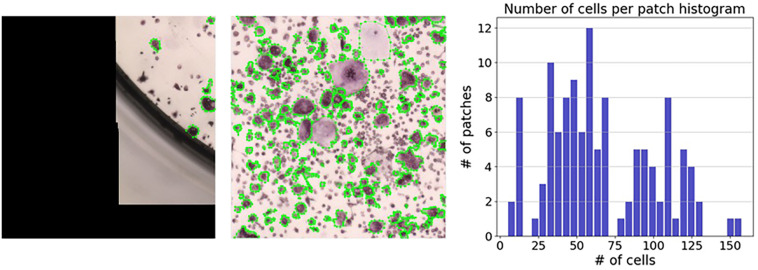
Distribution of the number of cells per region. The number of cells per region ranged from 5 annotated cells (left) to 158 annotated cells (middle). The right panel shows a histogram representing the number of cells per region.

For the annotation process, regions without any cells were discarded; this resulted in 133 regions with an average of 66.4 cells per region from 11 wells. Manually annotating a region took between several minutes for regions with few cells, and over an hour for regions with more than 100 cells (see Figure 7). Among the 133 regions we annotated, 27 regions had more than 100 cells (Figure 7 [right]).

### Patch Generation Strategy

Deep learning algorithms typically require a large number of examples for effective training. The considerations and challenges in annotating cell cultures described in the section “Data Collection” restrict the number of fully labeled cultures available. Thus, our main challenge was to train a deep neural network on relatively few training samples. A major advance made here stems from the realization that, in contrast to “images in the wild” where a single object can span most of the image, images of cultured cells can be viewed at a wide range of resolutions and preserve their semantic properties due to the small size of each cell compared to the entire culture. For example, when estimating the number of cells and the area covered by cells, we can restrict ourselves to small sections of the culture at any given point of time and still produce a perfectly annotated culture. This is not true when objects extend over a large section of the image such as in Figure 3.

Motivated by this observation, our strategy was to operate in small patch levels of $\frac{1}{8}\times \frac{1}{8}$, which means that each culture comprises 64 non-overlapping patches. This strategy also provides many different options for sampling (overlapping) patches and offers a substantial increase in the number of samples the model can be trained on. Specifically, we sampled a random region of the culture with 10 to 15% of the original width and height. The upper-left coordinate of the region defines the patch location. This process generates an enormous number of options for selecting a patch from a given culture. For example, given an image of a culture of size 1,000 × 1,000, there are more than 850^2^ ⋅ 50^2^ (≈1.8 ⋅ 10^9^) different ways to sample a patch by this procedure. Here, we sampled 3,600,000 random patches from 10 wells used for training.

Another mechanism for increasing the size of training data, is to apply non-informative transformations to training points. For example, adding a small amount of noise to an image is unlikely to change the semantic content, but provides a new data point for the algorithm to train on. In the deep-learning literature, this is known as “Data Augmentation” (Shorten and Khoshgoftaar, 2019). Here, we augmented the training data by applying a combination of the following transformations: (i) random photometric distortions, affecting brightness, hue, and saturation, (ii) random vertical flips of the patches, and (iii) rotations of the patches by 90°, 180°, and 270°.

Training the model with only 10 images of complete cultures did not allow for meaningful image detection. In contrast, the above procedures for generating random patches significantly increased the size of the training data, and thus improved the learned model.

### Configuration and Hyperparameters

**FIGURE 8:**
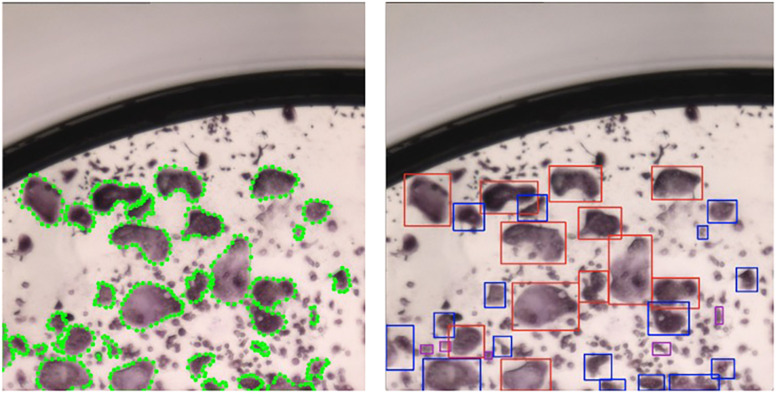
SSD implementation using bounding boxes. Left: patch with polygons annotated by a human annotator; Right: patch after transforming the polygons to the tightest square surrounding a polygon. Blue bounding boxes correspond to type I osteoclasts and red bounding boxes correspond to type II osteoclasts.

Our implementation was written in PyTorch, a deep learning library (Paszke et al., 2019) written in Python and C++. We used an open-source project as our starting point for SSD^1^. Since object detection pipelines were designed to detect bounding boxes, we transformed each polygon created during the annotation process to the tightest square containing the entire cell (Figure 8).

The implementation of SSD contains more than a dozen different hyperparameters, and we adapted some settings to fit our unique data. The main change involves reducing the number of convolution layers from 6 to 4, which removes bounding boxes spanning large sections of an input image. These objects are common in natural data (Figures 3, 4) and less relevant for culture well images (Figure 8). The modification of the architecture along with our strategy to operate on patches allows us to detect cells with sizes 51–383μm.

### Training

Because our scheme for augmentation extracts patches from full cultures, we dedicated 10 cultures for training, and 1 culture for testing. As is common practice for object detection, we utilized VGG16 (Simonyan and Zisserman, 2014), which was previously trained on ImageNet (Russakovsky et al., 2015) as the backbone network, and resumed training from these initial weights. We trained for 120,000 iterations with a batch size of 32 using an Adam optimizer (Kingma and Ba, 2014) with a learning rate of 0.00001.

### Detection in Large Images

As already discussed, our model was trained to detect cells in small patches. We therefore also needed a mechanism that could allow us to apply the model to images that are larger than these small patches. A simple strategy, which we employed here, is to divide the large image into a set of non-overlapping small patches and apply the model to each one individually. The output is then simply the union of the outputs from the small patches. Since our model was trained on $\frac{1}{8}\times \frac{1}{8}$ images, we had 64 patches per culture. It is true that splitting images into non-overlapping patches may split some cells across multiple patches and may therefore be considered a disadvantage (see “Error Analysis”). However, in practice, we found that the naive approach worked satisfactorily, and more sophisticated schemes were not necessary.

### Evaluation

**FIGURE 9:**
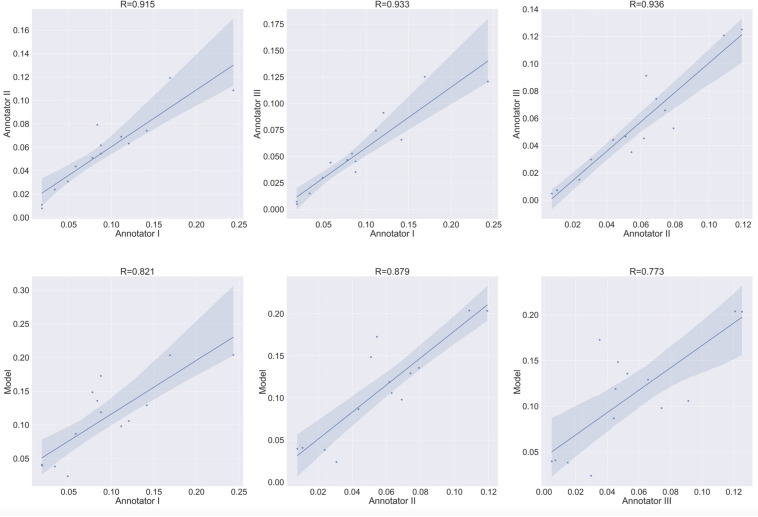
Agreement level on the cell counting task of Type 1 osteoclasts. Shaded area represents the variation boundaries. Upper row: correlation between the three annotators for the cell counting task performed on the same set of culture images. Bottom row: correlation between each annotator and the model’s prediction for the cell counting task.

**FIGURE 10:**
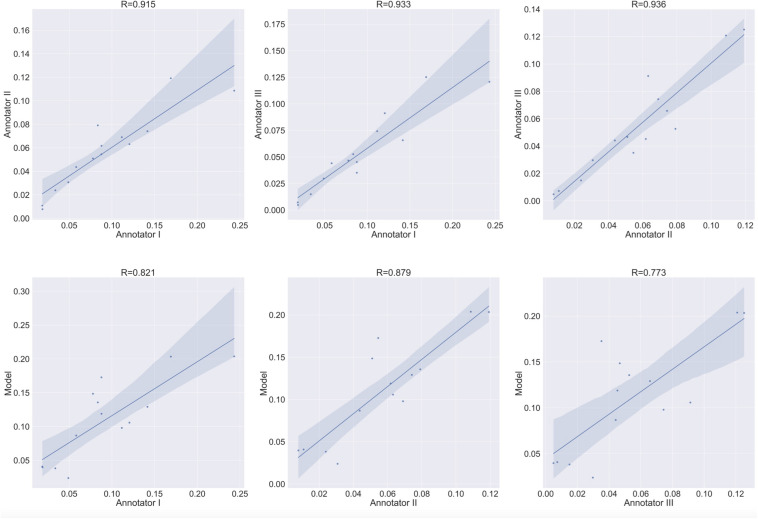
Area estimation task for osteoclasts Type 1. Upper row: correlation between each annotator on a single culture and the other two annotators on the area estimation task. Bottom row: correlation between each annotator and the model’s prediction on the area estimation task.

The main focus in bone cell culture analysis is to quantify the number and area of the cells rather than predicting the location of the cells inside the culture. Thus, the natural metrics for evaluation are the number of cells detected and the area covered by cells. Since these are inherently regression tasks, we report the Pearson correlation of (i) the human inter-annotator agreement for both the number and area covered by TRAP^+^ multinucleated osteoclasts (Figures 9, 10 upper rows), and (ii) the agreement between the model-predicted and human annotation for these measurements (Figures 9, 10 bottom rows). In these correlation analyses, the residuals were all normally distributed (as calculated using the D’Agostino & Pearson test in Prism 7.0, α = 0.05, *p* > 0.4).

## Results

### Inter Annotator Agreement

To verify meaningful annotations, we asked three annotators to annotate a test culture divided into 14 regions (the test culture consists of 16 regions, 2 regions did not contain any cells). We then evaluated the inter annotator agreement for the detection and analysis of preosteoclasts, Type 1 and 2 osteoclasts, and ghost cells (Figures 9, 10 upper rows, Tables 1, 2), which is an important measure of the accuracy and reproducibility of the labeling process. Because the most relevant osteoclastic cell type is a TRAP+ osteoclast with 3 to 15 nuclei (Type 1), it was important that our model discriminate between preosteoclasts and Type 1 cells as well as between Type 1 and Type 2 osteoclasts. The correlation between the 3 annotators was near-perfect for Type 1 and Type 2 osteoclast counting and area (>0.915), but more moderate coefficients were obtained for the detection of preosteoclasts (from 0.506 to 0.865). The discrepancies in annotation were mainly due to differences in the exhaustiveness of the labeling, which mostly affects the counting of small cells. Since the contribution of the small cells to the estimated area is low, there is still a high level of agreement for area measurements. Notably, the agreement among the human annotators on the identification of Ghost cells was generally low (0 to 0.917, Tables 1, 2).

**TABLE 1:** Correlation coefficients between human annotators and the model for cell counting.

	Annotator II	Annotator III	Model
**Correlation coefficients for preosteoclast numbers**			
Annotator I	0.506	0.819	0.879
Annotator II		0.687	0.730
Annotator III			0.920
**Correlation coefficients for Type 2 osteoclast numbers**			
Annotator I	0.952	0.955	0.968
Annotator II		0.960	0.913
Annotator III			0.921
**Correlation coefficients for Ghost cell numbers**			
Annotator I	0.597	0.287	0.269
Annotator II		0.781	0.410
Annotator III			0.423

**TABLE 2:** Correlation coefficients between human annotators and the model for cell area measurements.

	Annotator II	Annotator III	Model
**Correlation coefficients for preosteoclasts area**			
Annotator I	0.656	0.865	0.937
Annotator II		0.708	0.753
Annotator III			0.891
**Correlation coefficients for Type 2 osteoclasts area**			
Annotator I	0.980	0.978	0.995
Annotator II		0.991	0.978
Annotator III			0.978
**Correlation coefficients for Ghost cells area**			
Annotator I	0.326	−0.007	0.590
Annotator II		0.917	0.300
Annotator III			0.070

### Model vs. Human Annotation Agreement

Next, to assess the prediction accuracy of our model, we evaluated the agreement between our model and the three human annotators on the same set of images. These images were from a test set that was not used for training purposes. Overall, we found a high level of agreement between the model and human annotators (Figures 9, 10 bottom row, Tables 1, 2). When running our model on the same images used for the inter-annotator correlation, the model agreed with all 3 annotators with correlation coefficients >0.7 for preosteoclasts and Type 1 and 2 osteoclasts. Notably, the correlation among the three human annotators was similar and sometimes even inferior to the model-to-human correlation. In line with the inter-annotator low level of agreement on the identification of Ghost cells, the model’s correlation with the human annotators was also low (Tables 1, 2). For osteoclast (Type 1 and 2) counting, the correlation between the model and the human annotators ranged from 0.913 to 0.968, and for the area between 0.773 and 0.995 (Figures 9, 10 bottom row, Tables 1, 2). These correlation coefficients are comparable to the inter-annotator coefficients that ranged from 0.948 to 0.960, and from 0.915 to 0.991, respectively (Figures 9, 10 top row, Tables 1, 2).

### Error Analysis

**FIGURE 11:**
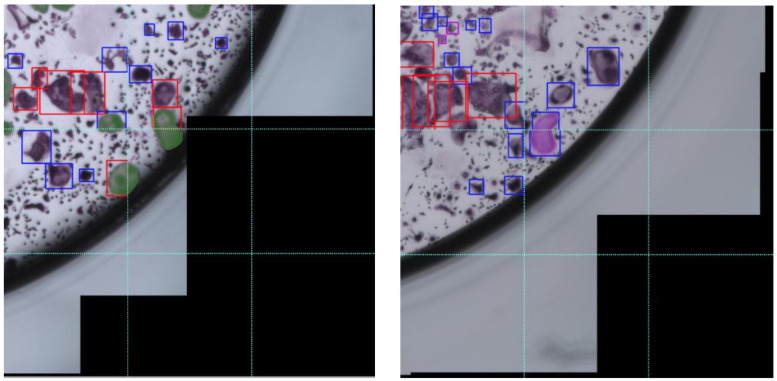
Error in annotation due to overlapping of cells over multiple patches. These two cropped images of cultures are divided into 9 smaller patches. Left: Cells that are split across different patches are marked in opaque green. Right: a single cell split across different patches is marked in opaque violet and is covered by two bounding boxes, one in the upper patch and one in the lower one.

In this section we describe the errors that our model makes. The most common issue is with cells that span multiple patches. This problem is introduced due to the resolution at which the model operates and is most frequently evident in bounding boxes that only partially cover the designated cells, as depicted in Figure 11 [left]. This type of error has a minor effect on the cell counting task, as most cells are covered by a single bounding box. In the area estimation task, this type of error causes partial coverage of the cells. In rare cases, a single cell is covered by two bounding boxes (See Figure 11 [right]), which causes double counting of a cell and has no effect on the area estimation task. A visual inspection of the predictions on the test images resulted in 15 erroneous cells from a total of 896 detected cells, which corresponds to 1.5% of the cells in the entire well. The impact of this type of error is therefore negligible.

## Discussion and Conclusion

We describe a novel method for rapidly and automatically quantifying osteoclast cultures that uses deep learning methods developed for object detection. Previously developed AI-based approaches for cell detection do not recognize the unique characteristics of osteoclasts and do not differentiate between cell types based on their nuclei number. For the training step, we manually annotated the type and location of each cell in 11 full cultures containing thousands of cells. The novelty of our strategy is that we train an SSD by working at “patch-level” instead of on the full culture and thereby generate more data for the algorithm to train on. The results indicate that our trained model is in good agreement with the human annotators, with a correlation that is similar to inter-annotator correlation. Our model performed especially well for the measurement of Type 1 and Type 2 osteoclast numbers and area. Measurements of preosteoclasts were slightly less satisfactory, although the model agreed with the human annotator to a similar degree and sometimes better than human annotators agreed among themselves. The identification of Ghost cells seems to be particularly problematic with little agreement among the human annotators or between the annotators and the model for these cells.

It should be noted that other experimental settings, e.g., using human cells, and different staining protocols, grayscale images, or camera resolutions, may require dedicated training of the algorithm. In such instances, we suggest that the use of the protocol reported here to generate large numbers of training images will provide a customized model that will perform satisfactorily in each specific setting. Using the same approach, the model could be further improved to detect subclasses of osteoclasts, i.e., 3 to 5 nuclei versus 6 to 10 and 11 to 15 nuclei. In theory, the model could also be trained to recognize and measure fluorescence-stained cultures.

In conclusion, we have developed a satisfactory method for the automation of osteoclast culture analysis that can detect and quantify TRAP-positive, multinucleated osteoclasts. This model discriminates between classical osteoclasts (3 to 15 nuclei) and abnormal “giant” cells (>15 nuclei). This model is therefore a useful labor-saving technique and we suggest that a similar approach may prove beneficial in facilitating other image related analysis tasks.

## Data Availability Statement

The raw data supporting the conclusions of this article will be made available by the authors, without undue reservation.

## Ethics Statement

Written informed consent was obtained from the individual for the publication of any potentially identifiable images or data included in this article.

## Author Contributions

EC-K, AB, SE, and ON: algorithm design and development. ZA: experiment design and development. ZA, MK, SBY, and HS: data curation. EC-K, ZA, YG, DN, AG, and YM: data analysis. EC-K, ZA, YG, DN, AG, and YM: writing. YG, DN, AG, and YM: study design and co-direction. All authors contributed to the article and approved the submitted version.

## Conflict of Interest

The authors declare that the research was conducted in the absence of any commercial or financial relationships that could be construed as a potential conflict of interest.

## Footnotes

1 https://github.com/amdegroot/ssd.pytorch
